# Effect of Surface Mechanical Treatments on the Microstructure-Property-Performance of Engineering Alloys

**DOI:** 10.3390/ma12162503

**Published:** 2019-08-07

**Authors:** Dharmesh Kumar, Sridhar Idapalapati, Wei Wang, Srikanth Narasimalu

**Affiliations:** 1 School of Mechanical and Aerospace Engineering, Nanyang Technological University, 50 Nanyang Avenue, Singapore 639798, Singapore; 2 Advanced Remanufacturing and Technology Centre, Agency for Science, Technology and Research (A*STAR), 3 CleanTech Loop, Singapore 637143, Singapore; 3 Energy Research Institute, Nanyang Technological University, Singapore 639798, Singapore

**Keywords:** nickel-based superalloys, surface mechanical treatments, severe plastic deformation, microstructure, fatigue performance

## Abstract

Fatigue is a dominant failure mechanism of several engineering components. One technique for increasing the fatigue life is by inducing surface residual stress to inhibit crack initiation. In this review, a microstructural study under various bulk (such as severe plastic deformation) and surface mechanical treatments is detailed. The effect of individual microstructural feature, residual stress, and strain hardening on mechanical properties and fatigue crack mechanisms are discussed in detail with a focus on nickel-based superalloys. Attention is given to the gradient microstructure and interface boundary behavior for the mechanical performance. It is recommended that hybrid processes, such as shot peening (SP) followed by deep cold rolling (DCR), could enhance fatigue life. The technical and scientific understanding of microstructural features delineated here could be useful for developing materials for fatigue performance.

## 1. Introduction

The type of material and its physical, chemical, and mechanical properties determine the performance of a component during its period of operation. Every product is made from one type of material dictated by its performance properties. Examples of such materials include the following: nickel-based alloys used for aerospace applications due to their excellent combination of fatigue, creep, and corrosion/oxidation resistance [1]; biocompatible titanium alloys (Ti-6Al-4V), Co-Cr, and 316L stainless steel alloys used for biomedical hard-tissue replacement applications [2]; steels used for load-bearing structural applications due to their combination of strength, durability, and cost [3]; and magnesium alloys used for automotive applications owing to their high specific strength [4]. However, mechanical properties of engineering alloys may be dramatically compromised under severe operating conditions such as cyclic loading, vibrations [1], corrosive environments, and elevated temperatures [5]. These conditions may lead to premature failure of the components during the service life. Thus, there is a constant need to improve the fatigue performance of the materials as approximately 50% of engineering components failure is due to fatigue, which is probabilistic in nature [6]. Fatigue life can be divided into the number of cycles for crack initiation and its propagation, leading to the ultimate failure of the component. As a rule of thumb, a hard mirror-like surface finish through polishing, compressive surface residual stress, and elimination of stress concentration zones through design modifications improve the fatigue life.

A variety of surface mechanical treatments (SMTs) such as shot peening (SP) [7,8,9,10,11], laser shock peening (LSP) [12,13,14], deep cold rolling (DCR) [15,16,17,18,19], and vibro peening (VP) [6,20] have been developed to mitigate the fatigue failure on a range of critical mechanical components in various industries [1]. The SP treatment is widely used in aerospace industry as it is generally believed that the compressive residual stress (CRS) induced by shot peening may impede the crack initiation and propagation at the subsurface region, hence contributing to the enhanced fatigue performance [21]. Laser shock peening achieves an appreciable CRS at a much deeper depth for certain fatigue critical components. The DCR treatment is another alternative process that could achieve both CRS and deep penetration depth [18]. The strengthening and deformation mechanisms in the nickel-based superalloy, Udimet720Li, during deep cold rolling process were proposed in our previous study [22]. The existing limitations of these processes, such as inaccessibility to treat complex geometries and rough surface finish, always motivate industries to develop new processes. The VP process is a newly developed process which produces a better surface finish and comparable CRS profile than that of shot peened parts, as reported in our previous study [23]. However, knowledge is still limited to residual stress and strain hardening, whereas, the microstructural changes under various surface mechanical treatments and their influence on final fatigue performance are not fully understood.

**Figure 1:**
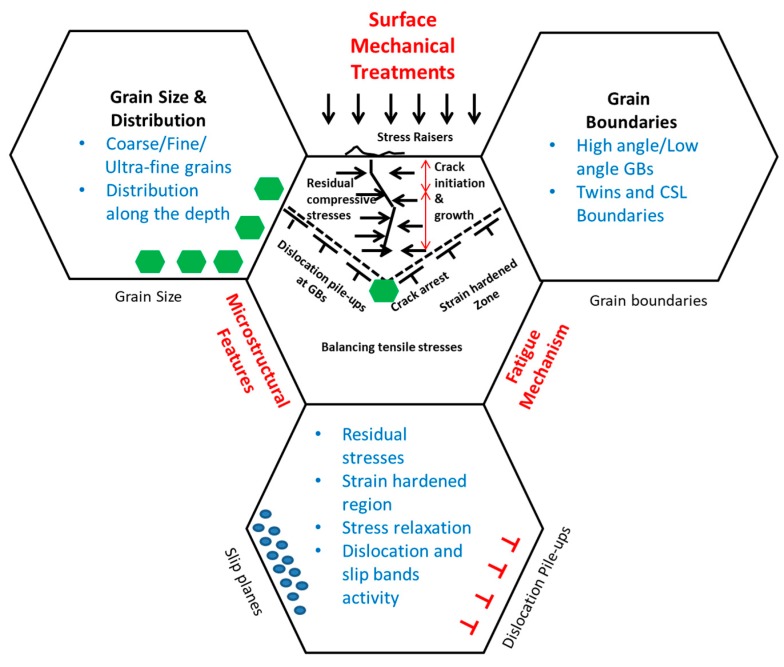
Schematic showing various microstructural features affecting fatigue mechanisms.

Microstructural features play a vital role in the fatigue behavior of polycrystalline materials. When tensile stress is present, surface/subsurface material imperfections could become stress concentration points and lead to crack initiation. This can be impeded by the introduction of CRS and strain hardening in the material after surface treatments (as shown in Figure 1). For the crack propagation, microstructural features and materials properties could have significant effects. For instance, grain boundaries impede the crack propagation by providing resistance to the crack tip, and provide strength to the material by impeding the motion of dislocations from one grain to another as it behaves as the pinning point. Therefore, a comprehensive understanding of fatigue damage due to microstructural features interacting is required to develop an optimized surface/subsurface material treatment process. This literature review focuses on the influence of residual stress and microstructural features on fatigue crack initiation and propagation. A variety of surface mechanical treatments are compared based on the process intensity, residual stress distribution, microstructure, mechanical property, and fatigue performance.

The first two sections cover the introduction and various aspects of fatigue failure, factors responsible for the failure, crack initiation, and propagation mechanism in polycrystalline engineering alloys. The second section provides a report on the fundamental microstructural features responsible for controlling fatigue. These features include the effect of grain size (coarse/fine/ultrafine/nanograin), grain distribution (homogeneous/gradient), interface boundaries (high/low angle/coherent/incoherent/twins), dislocation density, slip planes, residual stress distribution, and strain hardening on fatigue life. In the last two sections, the microstructural features generated through various process routes such as severe plastic deformation, surface mechanical treatments, and their effect on fatigue is investigated. Ultimately, a matrix is prepared for understanding the contribution of an individual microstructural parameter on mechanical properties and the fatigue performance. The main objective of this review is to provide a direction to optimize the fatigue performance of nickel-based superalloys through microstructural modifications for aero-engine components.

## 2. Fatigue Mechanism

Fatigue cracking is one of the critical failure mechanisms of structural components. Under cyclic loading conditions, a material often fails at a stress level below its nominal strength. The fatigue life of a component is presented as the number of loading cycles required to initiate a fatigue crack and to propagate the crack to a critical size. Therefore, fatigue failure occurs in two stages, crack initiation and crack propagation [24]. Fatigue cracks initiate from surface defects or regions of high-stress concentration, surface roughening generated due to vibrations or high repetitive stress amplitudes, and internal defects or material inhomogeneities.

**Figure 2:**
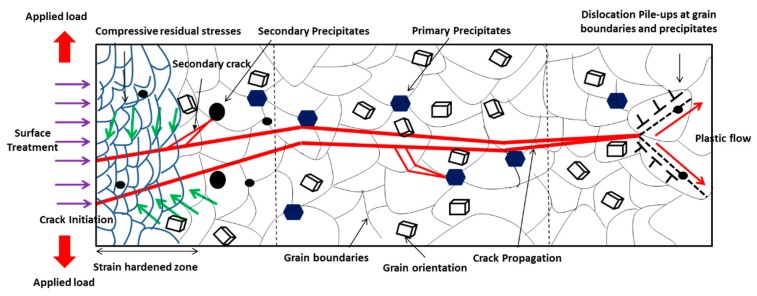
Schematic diagram showing interaction between crack propagation, precipitates, and microstructural features.

Fatigue is a stochastic process influenced by microstructure, surface topography, geometry, stress amplitude, frequency, and mean stress ratio, etc. Figure 2 shows the interaction of crack initiation and propagation with grain boundaries, dislocations, and other obstacles. These barriers, such as hard precipitates and grain boundaries, impede the propagation of a crack and improve the fatigue life [25]. It is important to understand crack initiation and propagation behavior and their interaction with microstructural features.

### 2.1. Crack Initiation Mechanism

Microstructural features, state of stress, materials properties, and surface topography are the critical factors in the crack initiation stage. Microstructural features such as dislocations, interface boundaries (i.e., grain boundaries, slip planes/bands, twin boundaries, and defects) are the primary sites for crack initiation [26]. It is observed that after a significant number of loading cycles, dislocations pile up creating persistent slip bands (PSB), which are the areas that rise above (extrusion) or fall below (intrusion) the surface of the component due to the movement of material along slip planes [27]. This leaves tiny steps on the surface that serve as stress risers where fatigue cracks are initiated. This is the first aspect of pure fatigue failure, and cracks approach in the region of the maximum dislocation activity in the active slip planes/bands [26]. Crack initiation is more prevalent in coarse grains where persistent slip bands (PSBs) are prone to generate. However, in small grains, the crack initiation is difficult due to the increment in the stress intensity threshold for nucleation. Under cyclic loading, dislocations pile up at the grain boundaries and induce stress concentration that initiates cracks to minimize the internal energy state. It means grain boundaries are a source of crack nucleation.

### 2.2. Crack Propagation Mechanism

The crack propagation mechanism is affected by microstructural features, precipitates, and compressive residual stress. It also depends on the threshold intensity factor below which fatigue crack propagation is not possible. Once the value of applied stress intensity is higher than the threshold, crack propagation starts in the grains and it propagates until a microstructural hindrance like a grain boundary, twin boundary, inclusions, or precipitates decelerates it. Therefore, grain refinement causes strengthening of the material by the insertion of microstructural barriers. Surface mechanical treatments contribute to the increase in some microstructural barriers per unit length due to the misorientation of grains. The different orientation of grains and grain boundary provides an obstruction to the propagation of the crack and simultaneously diffuses the crack [28]. Thus, these processes increase the fatigue life of the material significantly by generating various microstructural features, i.e., interface and dislocations.

In summary, the microstructural features such as grain size, interface boundaries, slip bands, and dislocations govern the crack initiation and propagation mechanism. Thus, further experimental investigation to improve the fundamental understanding of microstructure-damage mechanics is strongly required.

## 3. Influencing Factors of Ni-Based Alloy Fatigue Behavior

This section of the review covers the features that drive the fatigue crack initiation and propagation mechanism in the material.

### 3.1. Grain Size and Distribution

The effect of grain size, i.e., coarse (grain size more than 20 µm), fine (1–20 µm), ultrafine (between 100–1000 nm), nanocrystalline grains (less than 100 nm) on material properties is summarized here.

#### 3.1.1. Coarse, Fine, Ultrafine, and Nanocrystalline Grains

**Figure 3:**
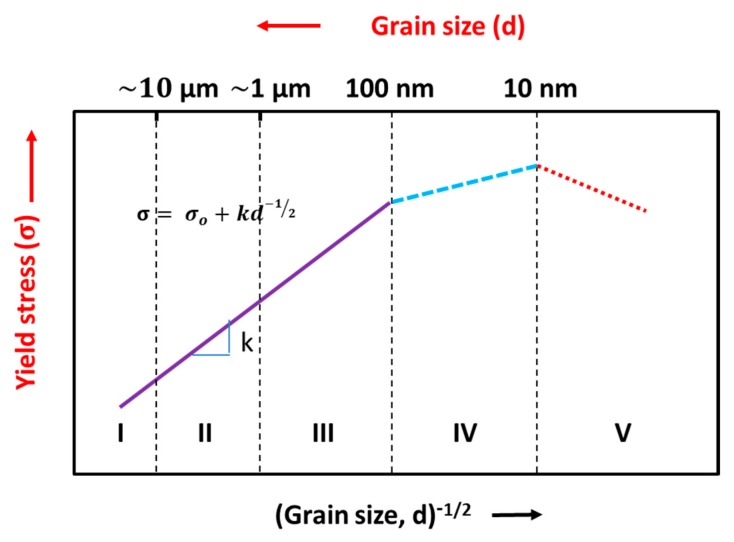
Schematic representation of the yield stress and grain size in microcrystalline, ultrafine grains and nanocrystalline metals and alloys (redrawn based on [29]).

Grain size is one of the most important factors to influence mechanical property and performance. The Hall–Petch relation predicts that as the grain size decreases the yield strength increases and it has been experimentally found to be an effective model for materials with grain sizes ranging from 1 mm to 1 micrometer [29]. For example, coarse grains (see stage I) exhibit low strength as the grain size is relatively large as shown in Figure 3. However, the ductility and toughness of coarse grains are relatively higher than fine grain structures. For fine and ultrafine grains (stage II and III), the decreasing grain size increases the strength at different rates at the expense of ductility. The mechanism involved in the rate decrement is still ambiguous. In stage IV for nanograins, it offers an ultra high-yield strength, fracture strength, lower elongation, toughness, and excellent wear resistance. However, the critical grain size and the depth of the nanocrystalline layer play a critical role in the enhancement of these properties. In stage IV, below 10 nm (typical critical value) materials soften due to grain boundary sliding [30] and follow the inverse Hall–Petch relationship which depicts that the saturation of grain refinement takes place up to a certain point, and then, further grain coarsening and dislocation annihilation mechanism takes place. This softening can be from various factors such as incomplete densification and atomic sliding at the grain boundaries [31].

The Hall–Petch relationship [32,33,34,35] is expressed as:

(1) $$\sigma ={\;\sigma }_{o}+\frac{k}{\sqrt{d}}$$

where, σ is yield stress, $\sigma_{o}$ is lattice friction stress required to move individual dislocation, *k* is the stress intensity for plastic yielding across polycrystalline grain boundaries (constant), and *d* is the average grain size. As per Hall–Petch, smaller grain size increases the strength through increased grain boundary area for blocking the dislocations, and hence there has been great interest in developing nanocrystalline materials [36]. Strengthening depends on the average grain size, grain size distribution, and grain boundary structure [37]. Strengthening is due to dislocation pile-up at the grain boundary that provides the resistance to deformation from structural refinement. In addition, it is also believed that the interactions of high- and low-range shear bands, which are developed by plastic deformation, contribute to the generation of subgrains, local dynamic recovery, and the recrystallization process that yields the grain refinement together with highly misoriented grains [38]. However, the grain refinement usually leads to a reduction in the ductility of the material. In contrast, plastic deformation by the dislocation glides can result in crystal rotation and a twist of grain boundaries (GBs) to improve the ductility of the material. Nanocrystalline structures are also notable for their mechanically unstable structure, since under high stress and deformation they undergo grain coarsening [39]. Maintaining both strength as well as ductility is practically difficult, however, the authors suggest a few methods to preserve the strength without the expense of ductility in the Section 3.1.2.

A material cannot develop high strength and ductility at the same time [40]. This is called the “paradox of strength and ductility” [41,42]. This loss of ductility at very small grain size comes from the low rate of strain hardening and strain sensitivity. During high strain, most of the dislocation boundaries induced by deformation develop into high angle grain boundaries and their frequency increases with the applied strain [43]. Thus, when the rate of strain hardening is high, dislocations accumulate at the grains which result in a reasonable level of ductility [44]. The formation of the ultrafine structure with non-equilibrium and high angle grain boundaries generates boundary sliding which maintains ductility in materials. Ultrafine crystalline structures with equiaxed grains and high angle grain boundaries impede the motion of dislocations, which enhances the strength of the material. Concurrently, these may facilitate another deformation mechanism, such as grain boundary sliding, and improve grain rotation which induces enhanced ductility [45].

**Figure 4:**
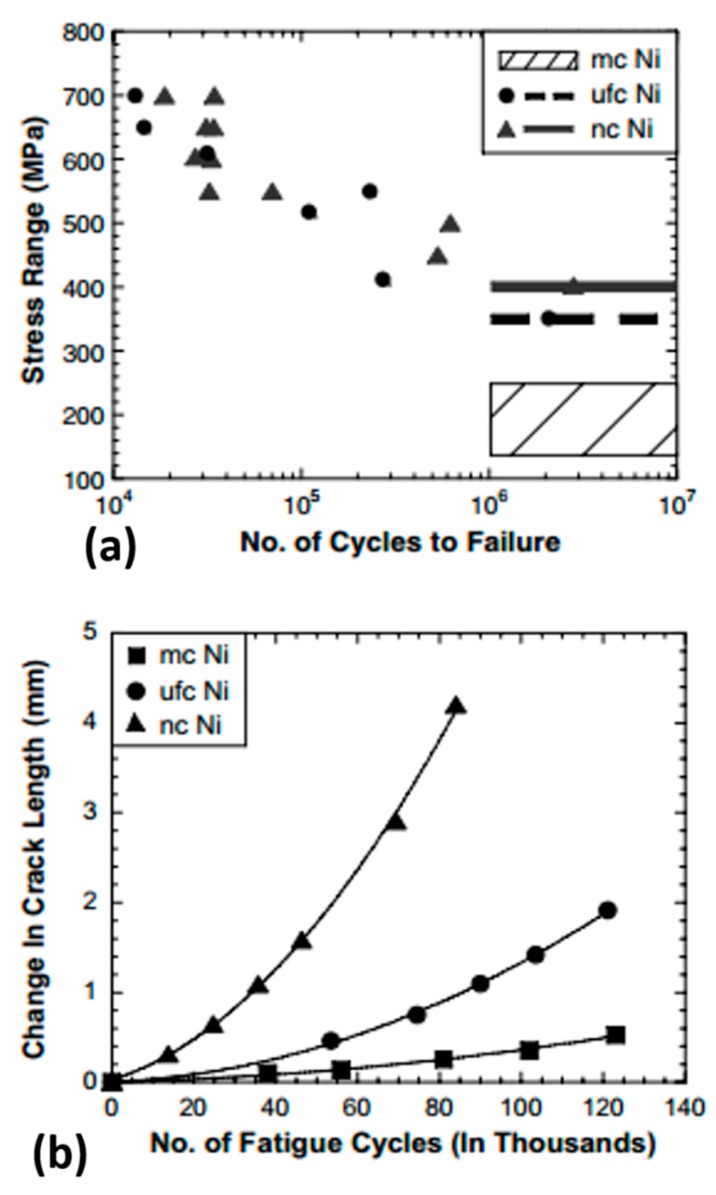
Comparison of (**a**) stress range and (**b**) crack growth in microcrystalline (MC), ultrafine grains (UFG) and nanocrystalline (NC) Ni at fixed stress intensity factor (adapted with permission from [48]).

It is accepted that the fatigue properties of materials are strongly dependent on their grain size [46]. Gayda et al. [47] reported on microcrystalline metals, where the grain size is typically greater than 1 µm, and found that an increase in grain size results in a reduction in the fatigue endurance limit. On the other hand, a coarser grain structure can lead to an increased fatigue threshold stress intensity factor, as well as a decrease in the rate of fatigue crack propagation. Conversely, grain size was found to govern the rate of crack growth under mechanical fatigue, with all other structural factors held constant. The microcrystalline Ni alloys measured a slower crack growth rate than that of nanocrystalline or ultrafine structures, as shown in Figure 4. The relevance of this pattern to microcrystalline, ultrafine-crystalline metals, and nanocrystalline metals is still unclear. Such lack of understanding is primarily a consequence of the paucity of experimental data on the fatigue behavior of metals with very fine grains.

Gayda et al. [47] described the temperature/grain size dependence of the crack growth behavior. At a lower temperature (T < 0.2 T_m_), reduction in grain size/crystallite size and irregularity of grain boundaries can improve the resistance towards short cracks propagation, but this resistance is lower for long cracks propagation. However, at the middle-temperature range (T ~ 0.4 T_m_), smaller grain size prompts intergranular fatigue fracture in the presence of high grain boundaries density due to the reduction in ductility [49].

#### 3.1.2. Gradient and Homogenous Structure

**Figure 5:**
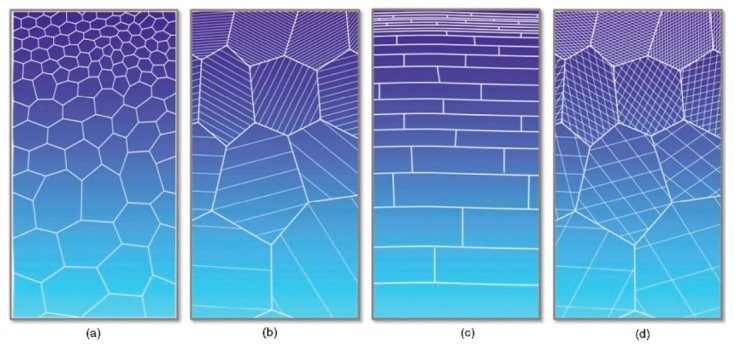
Gradient nanostructures with (**a**) grain size gradient, (**b**) twin thickness gradient, (**c**) lamellar thickness gradient, and (**d**) columnar size gradient (adapted with permission from [52]).

Gradient microstructures (GS) with a grain size gradient have been recently introduced to optimize the mechanical properties of structural materials [50]. It is reported that multiscale grain-size structures can be achieved by gradient plastic deformation processes such as surface impacting, grinding, and rolling. Gradient microstructures could obtain the trade-off between strength and ductility; both are mutually exclusive properties. It has been proven that gradient grain-size structures yield superior properties to homogenous structures [51]. According to reported results, the superior property is the significance of the optimum thickness of the GS layer (volume %), but it is still challenging to quantify the optimum thickness of the GS layer [52]. The gradient nanostructure can be further classified into the following four categories: (a) grain size gradient, (b) twin thickness gradient, (c) lamellar thickness gradient, and (d) columnar size, as shown in Figure 5.

**Figure 6:**
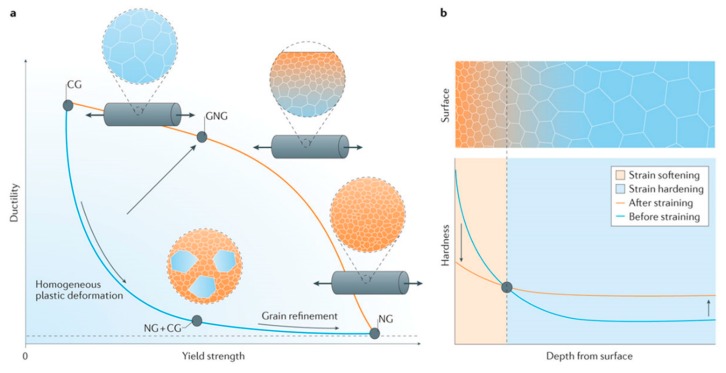
Representation of (**a**) ductility-strength synergy and (**b**) strain softening and hardening in gradient nanograined structures (adapted with permission from [54]).

It should be noted that the concept of gradient nanostructure, here, is very different from the strain gradient plasticity [52]. First, the range of the grain size gradient should be in the order of approximately, or more than, hundred grains, which is far less than other reported results of strain gradient plasticity mechanism [53]. Secondly, the applied strain throughout the depth is the same, unlike the strain variance throughout the depth in its counterpart. Lastly, the grain size should vary from the surface to the depth of the material (as shown in Figure 6), which results in varying yield stress in the material, and the applied strain is the same throughout the depth.

Lu [53] and Liu et al. [55] have employed a surface mechanical grinding treatment (SMGT) for bulk copper and nickel to produce strong, as well as tough, microstructures [56]. In this method, the surface of the material is under shear force, and the core of the material is not subjected to significant loading. Hence, surface layers produce nanograins, twins/twin boundaries, and slip planes/bands in the presence of shear force without altering the core microstructure. The topmost layers, which comprise the nanograins, provide strengthening to the material due to dislocation slips and accumulation of the coarse grains in the core, providing ductility or toughness. This means the overall material softening and hardening is taking place simultaneously creating strong and tough material. Furthermore, the synergy achieved by the grain size gradient is due to the gradient yield stress and mechanical incompatibility caused by a mismatch of the Poisson’s ratio in the outer plastic and inner elastic core. For example, the nanograin structure throughout the material depth provides strengthening and the coarse grain structure alone provides toughness and ductility, as shown in Figure 6. It is to be noted, as shown in Figure 6, that a microstructure with coarse grain embedded in the nanosize grains due to the homogeneous plastic deformation provides both low strength and ductility. The generation of bimodal grain size is also reported to be a method for generating high strength and ductility at the same time [57]. The nanocrystalline structure provides the strength, and embedded large grains stabilize the tensile deformation of the material [58]. It is to be noted that the gradient nanocrystalline structure (GNG) improves the fatigue life, whereas, the surface layer enhances the fatigue crack initiation threshold and coarse grains deflect the propagation paths of fatigue cracks by grain boundaries, thus, introducing crack closure and decreasing the rate of crack growth [59].

Thus, this synergy concept between two mutually exclusive properties, such as strength and ductility, together with high fatigue life, can be achieved through a gradient microstructure and a bimodal structure, which can be a potential application in aerospace and structural component designs [51].

### 3.2. Interface Boundaries

Interface boundaries play a major role in altering the material properties and failure mechanics. Factors that induce failure, such as crack nucleation and propagation, directly interact through the boundaries between grains [60]. Therefore, the behavior of the boundary affects the resistance toward its movement into another grain [52]. The geometry of the grain boundary could facilitate either slip transfer (in low angle GBs or twin boundaries) or resist the slip movement.

#### 3.2.1. Grain and Twin Boundaries

Twinning usually occurs as a coordinated movement of a large number of atoms together in a crystal by shearing action. This narrow misoriented region is considered as twin and the boundary that separates this region is termed the twin boundary. The major influencing factors for twinning are the plastic deformation, stacking fault energy, and grain size [61]. The face centred cubic (FCC) materials with low stacking fault energy deform by twins when subjected to plastic deformation. Further twinning can be observed in the materials with high stacking fault energy when subjected to a high strain rate [62]. Meanwhile, both high and low stacking fault energies are beneficial for the grain refinement process, since high stacking fault energy materials have a faster recovery rate and low stacking fault energy materials have twin formation [63]. Ultimately, stacking fault energy is the deciding factor for the twin formation as it influences the probability of cross slip. Cross slip and dislocation climb mechanisms are responsible for dynamic recovery [64,65]. Stacking fault energy can be modified through alloying in FCC metals [66]. At a specific temperature, the chance of twin grain formation increases if the stacking fault energy is low since dislocation annihilation is difficult [67]. For example, FCC metals with low stacking fault energies, such as Ag and Ni, usually deform by twinning [62,68,69,70,71], whereas, coarse-grained FCC metals have high stacking fault energy, and thus normally deform by dislocation slip.

Twinning is easier in the nanocrystalline materials as the grain size is in the 10 nm range, but it becomes difficult as the grain size further decreases [72,73,74]. Deformation twinning can be observed in the materials subjected to severe plastic deformation when the strain rate reaches a critical value [69]. Interestingly, in nanocrystalline material, thinner twins (<10 nm) play a major role in strengthening. In the nanometre range, twinning shows a strong resistance to the slip/slip bands. The persistent slip bands (PSB) twin boundary (TB) interaction (PSB–TB), can affect the fatigue response of the material.

**Figure 7:**
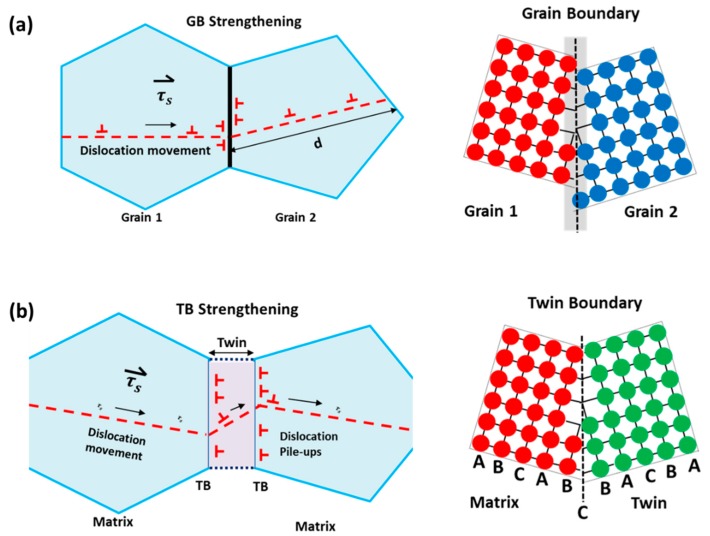
Schematic diagram of (**a**) grain boundaries and (**b**) twin boundaries (redrawn based on [70]).

Furthermore, nanoscale twin boundaries (CSL $\sum^{}3$) provide better resistance to the movement of dislocations and slip planes due to a narrow region of atomic mismatch and by decreasing their mean free path [71]. This atomic mismatch provides extra strengthening to the material as it induces dislocation pile-ups and hinders crack propagation, and thus exhibits high strength and thermal stability at elevated temperatures. Extreme segregation of dislocations on the twin/grain boundary induces stress concentration. To maintain the mechanical equilibrium, the position of this dislocation nucleation shifts to other grains or it can initiate cracks at the grain boundary. However, the resistance of dislocation movement through grain boundaries is low. Therefore, the dislocation pile-ups are rare in grain boundaries, as shown in Figure 7a, and no significant strengthening is noted. Twin boundary spacing, λ, is a critical feature that should be small for ultra-strong material properties, as shown in Figure 7b. The twin boundary increases the work hardening rate by acting as an obstacle for gliding dislocations [75]. Together, slip transfer at the boundary of nanoscale growth twins in FCC structures also provides the strengthening mechanism and ductility enhancement [76]. It has been demonstrated that the twinning mechanism can simultaneously maintain high strength and ductility of the material.

For the fracture mechanism, the twin boundaries play a vital role in crack initiation and propagation. Cracks usually initiate from the twin boundaries due to the concentration gradient generated by dislocation pile-ups and the twin boundaries impart higher resistance to crack propagation in the grains. The wide atomic mismatch between grains deflects the crack tip movement and makes its propagation difficult. However, there is always conflict among researchers with respect to the exact interaction mechanism of PSB-dislocation and twin boundaries.

#### 3.2.2. High Angle and Low Angle Grain Boundaries

**Figure 8:**
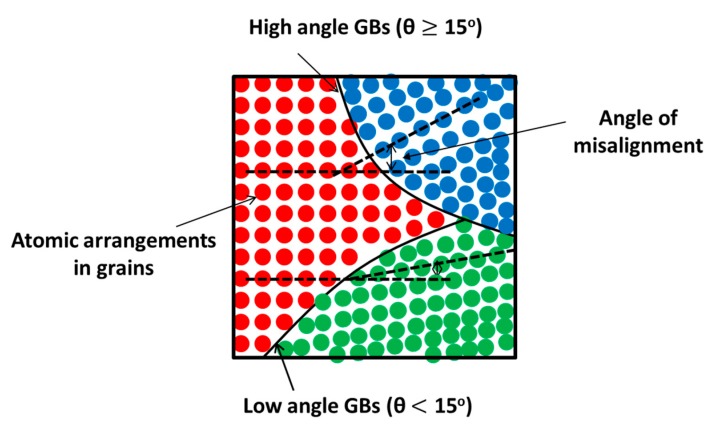
Representation of interface boundaries (redrawn based on [77]).

Interface boundaries are also categorized into high angle and low angle grain boundaries based on their angle of misalignment from neighboring grains. If the atomic misorientation angle between two grains is more than 15° (θ ≥ 15°), it is considered to be a high angle grain boundary, otherwise it is low angle grain boundary, as shown in Figure 8 [36]. High angle grain boundaries are generated through large accumulated strains (>6–8) in the material and help to arrest the cracks because they retard slip band formation and dislocation movement. Simultaneously, they induce more grain boundary sliding which yields high grain rotation and misorientation which ultimately results in high ductility and toughness of the material.

The formation of ultrafine grains with high angle nonequilibrium grain boundaries is very prone to sliding, which is not possible in low angle grain boundaries [57]. This results in a high ductility with high strength, fatigue, and toughness. The concept behind this mechanism is that the ultrafine grain possesses higher strength, and grain boundary sliding due to high angle nonequilibrium grain boundaries contributes to the higher ductility of the material.

Low angle grain boundaries are usually induced during plastic deformation which leads to strain hardening of the surface. They have poor grain boundary sliding, and thus result in high strain hardening but low ductility and toughness. In contrast, a few low angle grain boundaries (i.e., low angle twist or tilt grain boundaries with aligned or screw dislocations) do not effectively resist the dislocation movement, hence, they display lower strengthening [78].

#### 3.2.3. Coherent and Incoherent Grain Boundaries

**Figure 9:**
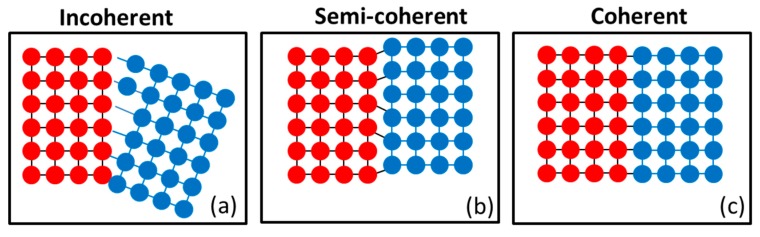
Schematic representation of interface boundaries (**a**) incoherent, (**b**) semi-coherent, and (**c**) coherent (redrawn based on [77]).

Coherent grain boundaries with atom positions on either side that are in proper contact, as in Figure 9, can be generated through physical and chemical processes such as electrodeposition, sputter deposition, plastic deformation, recrystallization, and phase transformation. These boundaries play a major role in the formation of a dense network of hard precipitates. For example, the precipitates γ, γ’, and intermetallics in Ni alloys are intended for strengthening at elevated temperatures. Several authors [70,79,80] have reported that nanoscale coherent boundaries are stable and generate strengthening effects while maintaining the toughness and ductility.

Furthermore, Seita et al. [81] conducted a study on the effect of hydrogen on fracture damage and found that coherent twin boundaries play a dual role in strengthening. Firstly, hydrogen embrittlement weakens the grain boundary leading to intergranular fracture (IGF) in coherent twin boundaries (CTBs). Crack initiation results from the localization of dislocations along persistent slip bands. However, hydrogen is known to enhance dislocation density in the material as compared to another deformation process. Researchers have reported that high dislocation density results in high strength and hardness, therefore, high fatigue life is anticipated but results are contrasted. Secondly, the resistance to crack propagation is governed by a completely distinct physical mechanism as the crack interacts with dislocation; it does not provide the medium to proceed through the next grain, so crack arrest takes place at the dislocation site [81].

Incoherent/semi-coherent grain boundaries do not create close crystallographic registry between grains and atoms separated by interface boundaries, as shown in Figure 9. A high fraction of incoherent grain boundaries obstructs the dislocation movement, thus, it significantly increases the strength and hardness, but it decreases the ductility of the material. This makes the material difficult to deform further as there is no scope for the accommodation of dislocations. In major structural applications, internal coherent boundaries with low excess energies was introduced as the main strengthening mechanism [70].

### 3.3. Dislocation Generation and Pile-Ups

The generation of dislocations and their movements significantly contribute to the strengthening of a material. Dislocation movement is the first aspect of fatigue crack initiation. In addition, dislocation pile-up at grain boundaries usually promotes large cracks to propagate further in the material. During plastic deformation, the energy provided to the material is converted to heat that raises the temperature of the material. A small fraction of this energy stored in the material itself causes lattice defects in the material, i.e., mostly dislocations. Grain boundaries are considered as the dense arrangement of tangled dislocations, which contribute to stored strain energy. Under external loads, dislocation movement and its segregation results in gross plastic deformation. Rupture and reformation of the interatomic bonds are the fundamental mechanisms for dislocation motion [82,83,84,85,86,87]. The nature of dislocation movement depends on the dislocation type, i.e., edge/screw. Edge dislocation can be moved by slip and climb, while screw dislocation can be moved by slip and cross slip. During the plastic deformation, movement and interaction of existing dislocations take place, which are very complex phenomena as the number of dislocations are moving on some of the slip planes in various directions. The hindrance occurs during the movements due to vacancy, grain boundary, defects, and surface irregularity. A higher critical resolved stress for dislocations is required to move further into the grains. This additional stress which is needed for the movement provides the strengthening of the material [88]. The hardness of the material is related to the dislocation density by the following equation [89]:

(2) $$H=H^{*}\;+\alpha Gb\sqrt{\rho }$$

where, *H* is microhardness, $H^{*},\;\alpha ,\mathrm{and}\;G$ are the material constants, b is burger vector, and ρ is dislocation density.

Alternatively, single weak grains are primary sites which activate the dislocation source and grain boundaries for dislocation pile-ups under stress, that induce stress concentration. However, piled up dislocations (only a few hundred) are not sufficient to form macroscopically observable extrusions and intrusions. The stress concentration in weak grains relaxes when the dislocation source moves to the adjacent grains and dislocation slips continuously form the nucleus of a fatigue crack of sufficient dimension. The mechanism for the formation of a fatigue crack source and the yield process are very similar to each other.

**Figure 10:**
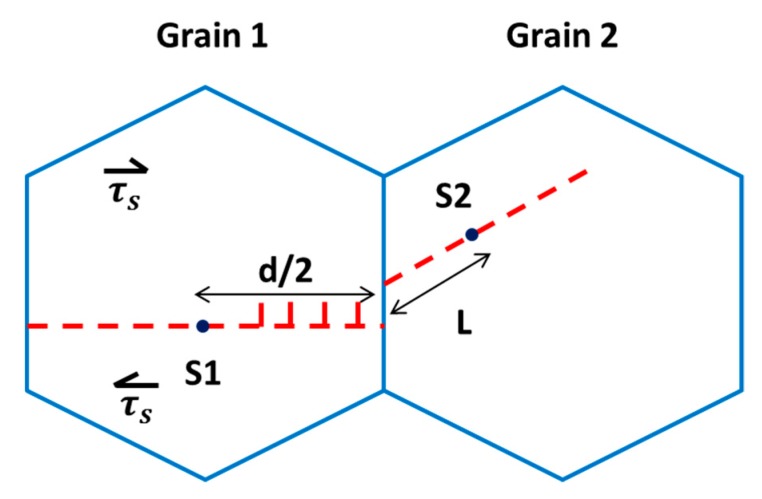
Pile-up formed in grain 1 under an applied resolved shear stress τs. S2 is a source in grain 2. A dashed line marks the trace of the preferred slip plane in each grain (redrawn based on [90]).

According to the Hall–Petch relationship, the yield strength is dependent on grain size of the dislocation pile-up mechanism. Yielding is first initiated from a single grain once a dislocation source becomes active and emits a dislocation loop. This dislocation loop then moves to the grain boundary, which resists further movement and results in a pile-up of dislocations. If the number of dislocations piled up at GBs is larger than geometrically required, then slip in grain 2 occurs immediately. The number of geometrically necessary dislocations before yielding is expressed as:

(3) $$n=\frac{\alpha \pi \tau_{s}d}{4\;Gb}$$

where, α is a constant near unity, $\tau_{s}$ is the average resolved shear stress in the slip plane, *G* is the shear modulus, *d* is the grain diameter, and *b* is the burger vector length. This accumulation of dislocations at the grain boundary results in stress concentration and if this stress concentration is effective, a source in neighboring grains will be activated to emit a dislocation loop (Figure 10). This stress concentration in other grains starts plastic deformation and this phenomenon extends further to the whole material [90].

According to the dislocation theory [91,92,93], as the particle size decreases the value of the critical shear stress for dislocation nucleation increases suddenly. The critical shear stress required for dislocation nucleation with the approximation that source size is equal to particle size is as follows:

(4) $$\tau_{f}=\frac{2aGb_{f}}{D}$$

and that required to nucleate the partial dislocation is:

(5) $$\tau_{f}=\frac{2aGb_{f}}{D}+\frac{\gamma }{b_{p}}$$

where, *b_f_* and *b_p_* are the burgers vectors of the full and partial dislocations, respectively, *G* is the shear modulus, and γ is the stacking fault energy. The factor *a* is taken as 0.5 for edge dislocation and 1.5 for screw dislocations.

Conversely, this dislocation pile-up theory is valid for pure metals, therefore, there is an alternate theory of grain boundary source for understanding the mechanism. According to the model of the grain boundary source, a grain boundary acts as the source of dislocation and the capacity to emit the dislocation depends on the character of the grain boundary. This theory is still under investigation and further study is required for the enhanced understanding of the mechanism.

### 3.4. Slip Band and Slip Planes

**Figure 11:**
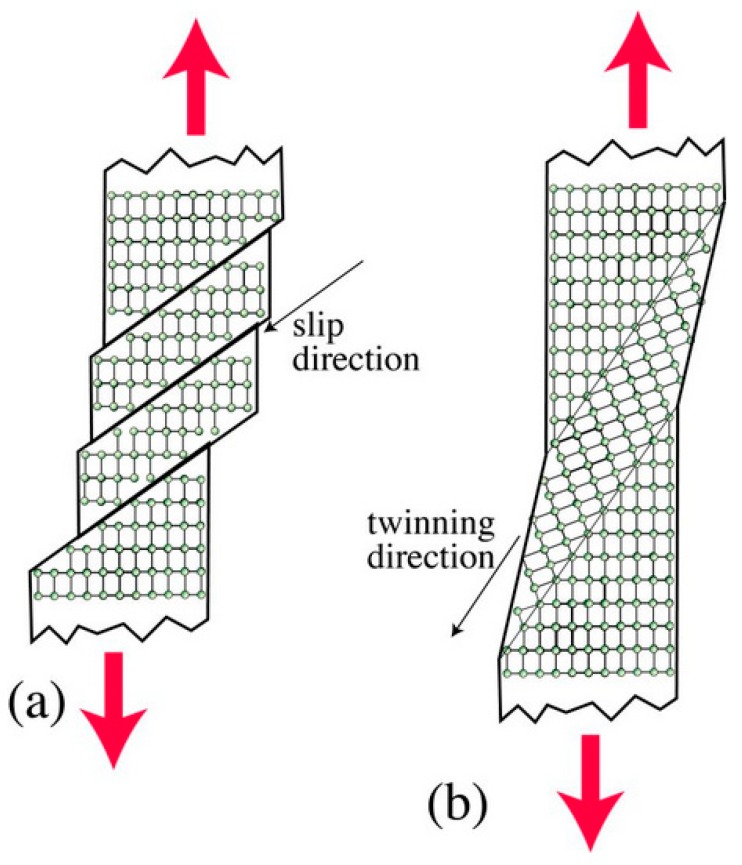
Differentiation between slip (**a**) and twin (**b**) interface (adapted from DoITPoMS/University of Cambridge). Available on: https://www.doitpoms.ac.uk/ldplib/shape_memory/background.php, accessed date (6 August 2019) [96].

The slip in crystal planes is the prominent mechanism of plastic deformation, which involves atomic sliding on different planes called slip planes, as shown in Figure 11. Slip occurs when the applied shear stress value exceeds the critical limit for the atomic movement [94]. Coherent slip band formation takes place at grain boundaries because of a pile-up of dislocations at the site [36]. These obstacles generate microstress concentration or discontinuity on the surface, encouraging nucleation of fatigue cracks at the stress raised site. Furthermore, cyclic loading that causes dislocations to move back and forth results in weakening of slip bands. This repetitive process weakens the point where it is fractured, i.e., shear decohesion which yields crack initiation [95]. Thus, slip plane characteristics, pile-ups, leads to the slip band crack initiation and propagation.

### 3.5. Strain Hardening Effects

Strain hardening is the strengthening of the material because of dislocation generation, multiplication, and their movement due to plastic deformation. After strain hardening, the saturation of plastic deformation takes place, as it cannot accommodate further dislocation generation, and provides resistance to the deformation. This resistance to deformation comes from the obstacle structure in the material, which controls the movement of the mobile dislocations. Thus, it increases the yield strength (τ), hardness, and tensile strength of the material. However, dislocation annihilation and residual stress relaxation takes place due to atomic vibrations at the higher internal energy generated due to a high temperature condition [97,98,99,100,101]. Hence, the magnitude and rate of stress relaxation depends on the degree of strain hardening developed in the material [102].

Strain hardening is also responsible for enhanced resistance to crack initiation due to surface/subsurface strengthening. On the other hand, it causes lower crack growth resistance due to material embrittlement [103], i.e., lower ductility in aluminum. Strain hardening or work strengthening is the significance of dislocation arrest by grain boundary or any other obstacle. As reported by Guechichi et al. [104], the effect of strain hardening on the fatigue life improvement of the material is more crucial than compressive residual stress. Almost half of the residual stress is relaxed during the first few fatigue cycles in torsion, rotatory bending, and the tension-compression test. However, strain hardening does not change appreciably and is relatively more stable even at elevated temperatures. Overall, there is ambiguity over the dominance of compressive residual stresses, strain hardening or plastic deformation on the fatigue performance. It is believed that the improvement is attributed to complex interactions of all factors such as material properties, microstructure, and topography [105].

### 3.6. Compressive Residual Stress Distribution

The generation of the compressive residual stress (CRS) fields in the surface or subsurface layers of the material are well known for fatigue life improvement. Strain hardening, CRS, surface finish, and phase composition are the influencing factors for the fatigue failure mechanism [106]. The crack initiation could be due to tensile stress present on the surface or subsurface, and therefore the introduction of CRS can compensate for these adverse effects as both are opposite in nature. CRS resists the surface crack initiation and could even force subsurface initiation, i.e., the tensile region in the subsurface [107]. It has been reported that shallow residual stress field is enough to impede the crack initiation from the surface and higher fatigue endurance can be achieved if the crack source is under the strain hardening zone [103]. CRS also plays an important role in impeding the crack growth, as the compressive stress field closes the crack tip to propagate further [108]. However, the individual dominance of magnitude and depth of CRS on fatigue crack initiation and propagation is not yet fully understood and needs further investigation.

At elevated temperatures (T > 0.4 T_m_), the stress relaxation is a major concern that eliminates the beneficial effect of compressive stress induced in the material due to material annealing. Overall, the beneficial effect on fatigue life is pronounced due to the complex interactions of strain hardening, hardness, compressive residual stress, and dense dislocation structure [109].

## 4. Severe Plastic Deformation Techniques for Bulk

Microstructural tailoring during bulk material fabrication can provide superior properties [110] as these are nanostructural features such as nanograins, nanotwins, and nanoclusters [111]. In this section, different methods for generating tailored microstructures are discussed.

### 4.1. Equal Channel Angular (ECA)

**Figure 12:**
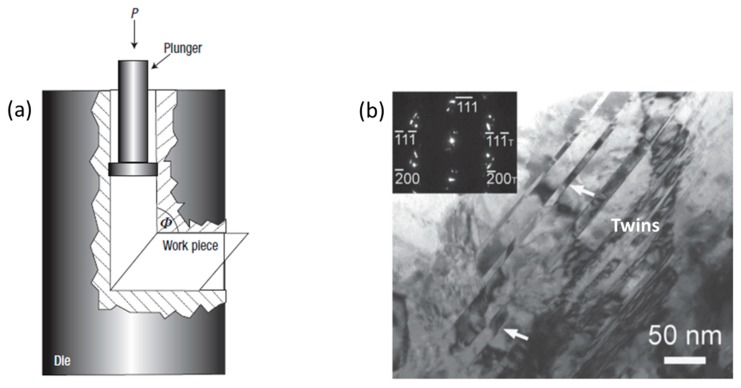
(**a**) Schematic diagram of the equal channel angular (ECA) process and (**b**) Bright Field-TEM image of a grain with high deformation twins (adapted with permission from [57,110]).

Equal channel angular (ECA) pressing is an effective method (Figure 12a) for producing nanostructured material with a combination of excellent materials and structural properties [112]. A few researchers have reported on the following varieties of interface boundary: (1) low/high angle GBs, (2) special and random GBs, and (3) equilibrium/nonequilibrium GBs. Because nanograined materials are highly controlled by interface structure, therefore, they display different material properties under various interfaces. Nanograins and nanotwins (Figure 12b) that are generated by a severe plastic deformation process improve the strength and ductility simultaneously [36]. The favorable conditions for generation of nanotwins are the following: (1) relatively low stacking fault energy, (2) low deformation temperature, and (3) high strain rate [73].

**Figure 13:**
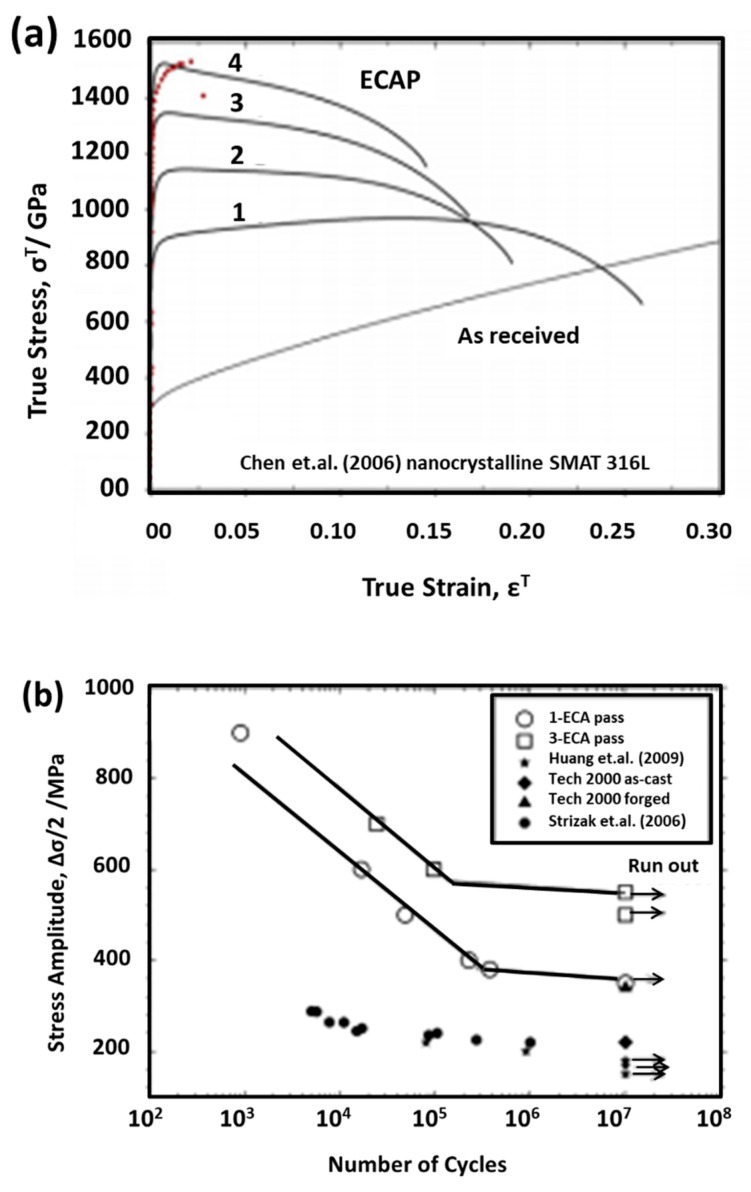
(**a**) Tensile properties and (**b**) Stress amplitude-number of cycles (S-N) curve for high cycle fatigue performance of 316L steel (adapted with permission from [113]).

Ueno et al. [113] reported significant improvement in tensile strength (Figure 13a), high cycle fatigue (HCF) (Figure 13b), and fatigue endurance limit in nanostructured 316L stainless steel without compromising ductility. This improvement was attributed to the high fraction of nanotwins and high stability of twin boundaries. Furthermore, Vinogadrov et al. [114] investigated the effect of strain path during the ECA process on grain refinement and structural features for Cu-Cr alloys. The grain shape and texture effect on ductility and strain localization was observed, hence, it could influence the cyclic loading behavior.

However, there are certain limitations of this process as it can be applied to only small/thin discs. The components with relatively large and complex geometries are still challenging to process using this method.

### 4.2. High-Pressure Torsion (HPT)

**Figure 14:**
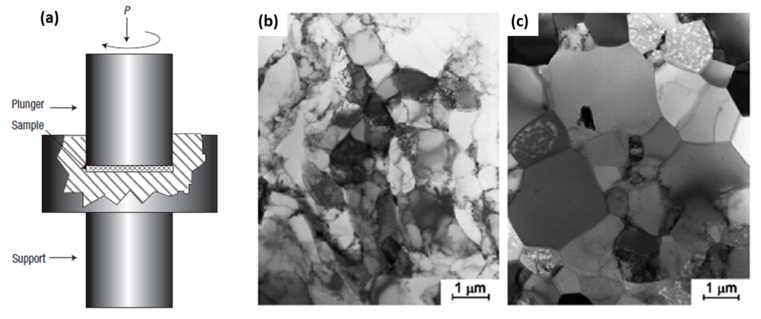
(**a**) Schematic diagram of the high-pressure torsion (HPT) process, and TEM micrograph of aluminum (Al) disc at 1.25 GPa, (**b**) at the center, and (**c**) at the edge (adapted with permission from [57,117]).

High-pressure torsion (HPT) (shown in Figure 14a) can generate an ultra-fine crystallites (UFC)/nanocrystalline structure with excellent mechanical properties through combined compressive force and torsional straining [115]. This is attributed to a large fraction of high angle grain boundaries with high internal stress in Ti-6Al-4V [116]. Zhilyaev et al. [117] reported the high fraction of high angle grain boundaries (HAGBs) (~68.1%) as compared with ECP (~60%) for nickel alloy with an average grain size of ~0.27 µm.

However, Zhilyaev et al. [118] reported significant grain refinement and hardness increment in pure nickel. A homogeneous microstructure with a large fraction of low angle grain boundaries (LAGBs), twins, special boundaries, and a small fraction of HAGBs was observed. Interestingly, grain size, interface boundaries, and material properties varied from the edge (Figure 14c) to the center of the circular disc (Figure 14b). The average grain size at the center was ~0.8μm and ~1.2μm at the edge as reported by Xu et al. [119] for aluminum (Al). High dislocations can be observed through the TEM micrographs (Figure 14b,c) which are not widely separated due to high stacking fault energy and there is easily cross slip.

Valiev et al. [57] used HPT to process pure Al (99.9%) disc of 10–20 mm diameter and 0.2–0.5 mm thickness under 6 GPa pressure. This produced a nanocrystalline structure with deformation twins, nonequilibrium grain boundaries, dislocations, and solute segregation. Vickers hardness and tensile strength of the material significantly increased.

**Figure 15:**
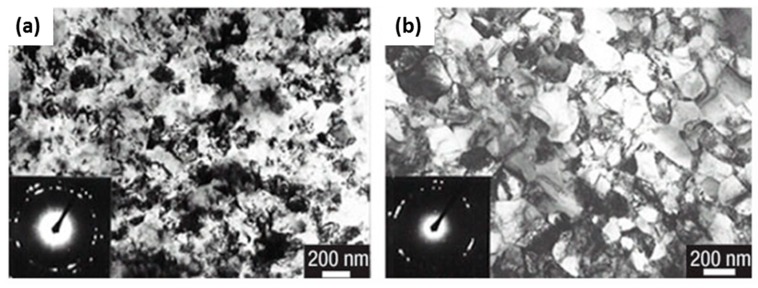
TEM micrographs of (**a**) copper processed by HPT (5 turns at 6 GPa) and (**b**) processed by ECA (12 passes) (adapted with permission from [57]).

**Figure 16:**
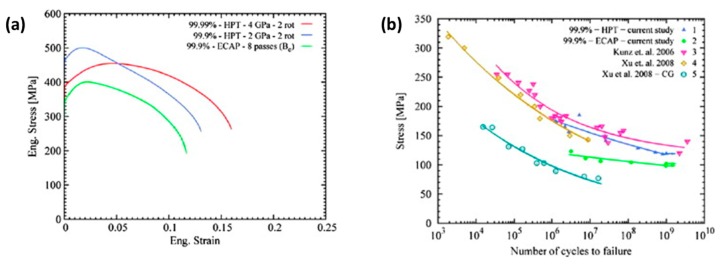
(**a**) Stress-strain curve and (**b**) S-N curve for HPT and ECP processes in copper (adapted with permission from [120]).

Commercially pure copper processed by HPT showed considerable improvement in fatigue performance of the material as compared to the ECA process. The nanograins, nanotwins, and dislocation density have more fraction of the components generated by HPT (Figure 15a) as compared to the ECA process (Figure 15b). However, the microstructure varied across the diameter of the disk processed by HPT. The uniaxial tensile stress-strain variations for the samples between these two processes show that HPT showed a remarkable improvement in strength (Figure 16a) and fatigue life (Figure 16b).

In conclusion, the microstructural architecture, such as bimodal grain structure/UFC structure with high angle and nonequilibrium grain boundaries, can provide improved material properties such as strength, hardness, ductility, fatigue, and wear resistance [121,122,123]. The slip band formation, substructure, and deformation phenomenon are also observed in copper during the ultrasonic vibration energy and the thermosonic energy bonding process which are being used in electronic applications. The wire bonding strength has been discussed based on the deformation mechanisms [124,125]. These could be a topic of great interest to several researchers and industries for developing next-generation components.

## 5. Surface Modification Techniques

The surface interacts with the surrounding environment and loads. Hence, it is more likely to deteriorate over time, for example, fretting, fatigue, corrosion, wear, and creep [126]. The surface characteristics of engineering materials have a significant effect on the serviceability and component life. Thus, it cannot be neglected in design. Surface modifications involve protective coating, cladding, heat treatment (e.g., nitriding and carburizing), and surface mechanical treatments. Surface coating [127,128] and deposition methods are not effective for altering the microstructure driven fatigue failure and are not discussed here (readers can refer elsewhere [129,130,131,132,133,134,135,136,137,138,139] for these processes). Surface mechanical treatments are the focus of this discourse.

Surface mechanical treatment is a method used to alter the surface or subsurface characteristics such as morphology, microstructure, and materials properties through plastic deformation. Modern turbine blades of jet engines are subjected to these surface treatments for improved fatigue performance [140]. The responsible mechanisms for plastic deformation are the slipping, twinning, and dislocation generation, which were described in the previous section.

Surface modifications usually generate the high-density nanocrystalline structure at the surface that attributes to the strengthening. This strengthening is the sum of contributions from dislocation strengthening and boundary strengthening, apart from frictional stress, as given by [32].

σ = σ_o_ + σ_ρ_ + σ_b_ (6)

where, *σ_o_* is the friction stress, σ*_ρ_* is the forest hardening, and *σ_b_* is the grain boundary hardening.

The dislocation in the low angle boundaries and the volume between the boundaries causes forest hardening. Grain boundary hardening is caused by the high angle boundaries, which is taken to be inversely proportional to the square root of boundary spacing [141].

Microstructural evolution can be understood by the mathematical expression which depicts the blend effect of strain rate and deformation temperature during plastic deformation which is expressed as the Zener–Hollomon parameter (Z) [67]. It does signify that the low deformation temperature and high strain rate can impede the dislocation annihilation mechanism, which will result in grain refinement by the formation of new grain boundaries generated through dislocations.

(7) $$Z=\;\dot{\varepsilon }\;\times \;e^{Q/RT}$$

where, *Q* is the activation energy mechanism controlling the rate of deformation, *T* is the absolute temperature, and *R* is the universal gas constant.

It is understood that strain rate, deformation temperature, plastic deformation, and strain gradient are the key factors for the surface modifications. Structural refinement and reduction of grain boundary spacing govern the dislocation multiplication which provides excellent strength and mechanical properties, although further grain refinement can lead to grain coarsening and dislocation annihilation due to grain boundary migration. Therefore, the high strain rate shear deformation can lead to higher dislocation multiplication with lower angle grain boundaries and lower grain size. This shear deformation also generates a strain gradient which is important for the creation of geometrically necessary dislocations [142,143].

**Figure 17:**
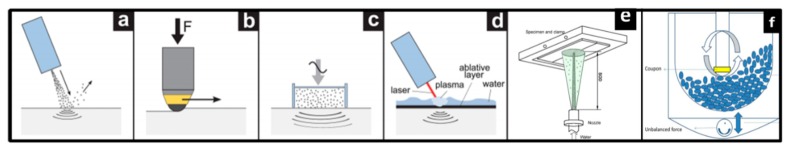
Surface mechanical treatments: (**a**) shot peening (SP), (**b**) deep cold rolling (DCR), (**c**) ultrasonic shot peening (USSP), (**d**) laser shock peening (LSP), (**e**) water jet cavitation peening (WJCP), and (**f**) vibro peening (VP) (**a**–**e**) adapted with permission from [144,145]).

Surface mechanical treatment (SMT) processes include shot peening, deep cold rolling, water jet cavitation peening, laser peening, and vibro peening, as shown in Figure 17, and are discussed below. Several results are reported in the nascent microstructural condition of the sample, but in the post surface treatment, microstructural evolution evidence is still limited. This means microstructural evolution after surface treatment and governing mechanism of fatigue crack damage requires further investigation.

### 5.1. Shot Peening (SP)

Shot peening (SP) is a surface mechanical treatment method in which bombarding of high-velocity shot media generates plastic deformation and induces compressive residual stress and strain hardening in the material. It could improve the fatigue life significantly by impeding the crack formation and propagation in the surface/subsurface [144]. However, several microstructural features also contribute to the fatigue life improvement.

Several researchers [10,107,146,147,148,149,150,151,152] are working on its effect on material properties, residual stress, strain hardening effect, dislocation density, slip planes, and grain boundary behavior. There are few results reported for the quantification of strain hardening effect, grain size distribution, and interface boundaries. Song et al. [153] reported the fraction of interface boundaries and its correlation with shot peening intensity for titanium alloys (TC21). In this work, the EBSD technique was used to quantify the fraction of low angle (θ < 15°) and high angle grain boundaries (θ ≥ 15°) based on the misorientation angle. The fraction of low angle grain boundaries was 0.59 and the fraction of high angle grain boundary was 0.38 from a 100 µm depth from the surface. Deep in the material (100–360 µm), the fraction of low angle grain boundary was 0.262, and the high angle grain boundary fraction was 0.72. The dislocation networks were composed of dislocation bands and slip bands in α phase of the material. There were no twins and stacking faults observed in the surface layer [153]. Shekhar et al. [154] studied the behavior of strain rate on the interface boundaries and refinement and quantified the volume fraction of low angle and high angle grain boundaries. The advanced characterization, such as EBSD/TEM characterization, can distinguish the fraction of twin boundaries and atomic misorientation or kernel average misorientation [155].

**Figure 18:**
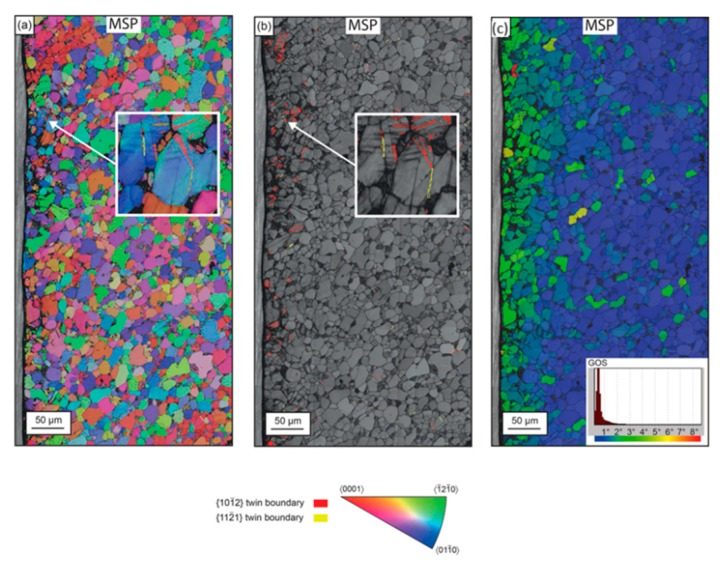
The electron backscattered diffraction (EBSD) image of treated surface (**a**) inverse pole figure image, (**b**) band contrast, and (**c**) grain orientation spread (adapted from [156]).

**Figure 19:**
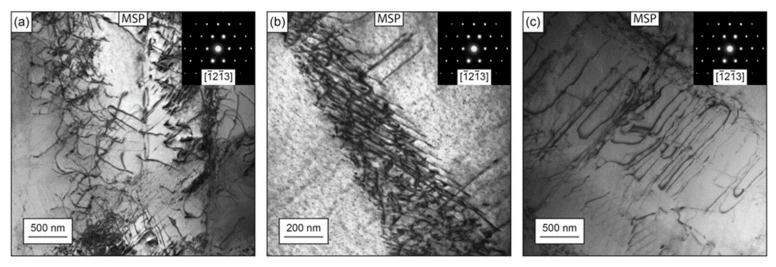
Bright field-TEM micrographs of shot peened area showing (**a**–**c**) dislocation structures (adapted from [156]).

Lainé et al. [156] studied the behavior of dislocation structure, deformation twinning, and crystallographic effects on metallic shot peened Ti-6Al-4V, as shown in Figure 18, and reported that MSP generates long wavy tangled dislocation structures and shear bands in the material due to a high-accumulated strain and strain rate (10^4^ s^−1^). The tangled dislocation structure was due to the localized work hardening from multiple subsequent shot impacts. The plastic deformation was in the form of deformation twins (Figure 18a,b) because it was very sensitive to grain size, which was higher for large grain size. Furthermore, the depth of strain hardening was quantified through the grain orientation spread (GOS) plot in EBSD, as shown in Figure 18c, which was 70 µm from the top surface. A correlation was also reported between residual stress depth generated through the GOS and hole drilling method. Altenberger et al. [157] also investigated the effect of near-surface microstructure generated by shot peening on the performance of stainless steel (AISI 304). A complex subsurface microstructure, consisting of nanocrystalline layers, deformation bands, and strain induced martensitic twin lamellae with high dislocation densities in the austenitic matrix was observed (Figure 19). Nanocrystalline surface layers provided stability against cyclic loading, even at high-stress amplitudes, and impeded dislocation movement and slip band formation. Interestingly, the depth rather than the intensity of stain hardening was found beneficial for fatigue life improvement. Later, Zhan et al. [158] investigated the effect of gradient microstructure generated by shot peening of steel (S30432). The microstrain, microhardness, and domain size varied along the depth and was observed through X-ray diffraction method.

It is understood that induction of compressive residual stress, strain hardening, dislocation generation, and grain refinement are beneficial after shot peening. Unfortunately, the benefits of shot peening may be reduced or even completely eliminated at a high operating temperature (T > 0.4 T_m_). Kim et al. [159] reported stress relaxation at an elevated temperature and isothermal fatigue reduced up to 50% at 650 °C to 725 °C for Udimat 720 Ni-based alloy. This relaxation occurs due to dislocation and diffusive movement of atoms that reduce the underlying misfit. At a short timescale at 350°, the mechanism of stress relaxation was due to the accelerated kinetics at the high stored energy level. At longer times, this mechanism was due to creep-related phenomena as it significantly reduces the work hardened zone. Interestingly, the effect of thermal exposure was very strong on the residual stress rather than depth of cold work in the measured temperature range. On the surface, the percentage cold work decreased instantly as the temperature increased beyond 650 °C [160], which shows the temperature dependence of mechanical properties [161].

Shot peening has been demonstrated as a fatigue life improvement process as reported by several researchers for steel [8,162,163,164,165,166], aluminum [9,11,14,167,168,169,170,171], magnesium [7,172], nickel [97,147,173,174,175], and titanium alloys [176,177,178,179,180,181,182,183,184,185,186,187,188] and as summarized or quantified in Table 1.

In conclusion, there is still limited knowledge with respect to the correlation of fatigue performance and microstructural features which require further investigation to engineer the material with optimum microstructural features. This work could extend to explore other surface enhancement techniques for optimum microstructural features development.

### 5.2. Vibro Peening (VP)

Vibro peening (VP) [6] is a hybrid technique of shot peening and vibro polishing, which generates a peened and polished surface as compared to other available techniques such as shot peening or laser peening. The use of an unbalanced motor vibrates media bowl, allowing it to flow against the surface. The kinetic energy generated during the vibration of media deforms the surface through shear and compression. The major fraction of the kinetic energy of media transferred perpendicular to the surface (compression) induce the plastic deformation and the other fraction as shear action induce burnishing [6]. This process induces compressive residual stress deep into the material and maintains roughness on the surface. This advanced technology is cost effective and reduces overall process time because it combines both mechanical treatment and polishing.

**Figure 20:**
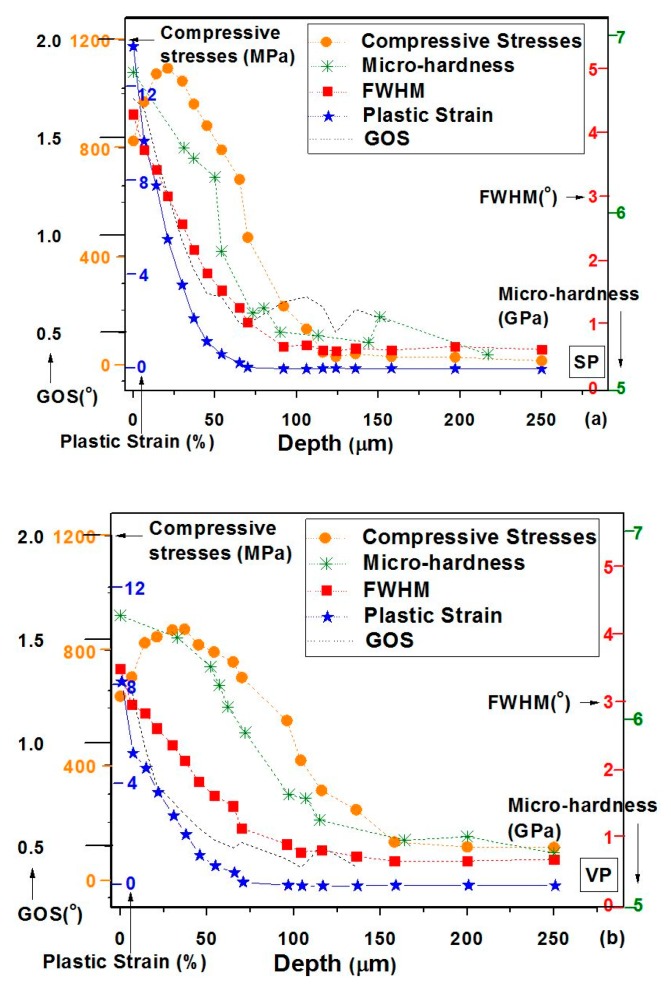
A comparison of residual stress, microhardness, full-width half maxima (FWHM), plastic strain, and grain orientation spread (GOS). (**a**) SP and (**b**) VP (adapted with permission from [23]).

Feldmann et al. [6] reported that the low surface roughness value (R_a_ ≤ 0.25 µm) can be achieved by vibro peening, cold rolling, and vibro finishing, but shot peening generates the high roughness value (R_a_ ≈ 0.65 µm) which is beyond the acceptable range of aerospace components. However, the compressive residual stress is lower in magnitude and shallower in depth in vibro peening (maximum 800 MPa at 50 µm) as compared with shot peening (maximum 1050 MPa at 100μm), deep cold rolling (maximum 780 MPa at 150 µm), and the combination of shot peening followed by vibro finishing (maximum 1050 MPa at 45 μm). Corresponding to these values, the HCF life of components has been increased by 32% in deep cold rolling, 35% in vibro peening, 61% in shot peening, and 66% in a combination of shot peening and vibro finishing in the IN718 HPC blisk aerofoils [5]. Ardi et al. [20] investigated the effect on residual stress, cold work, and surface integrity on RR1000 and found that a desirable compressive stress with low cold work could be generated for optimizing the fatigue performance of the components. The quantification of the depth of strain hardening was done by grain orientation spread (GOS) using an electron backscattered diffraction (EBSD) technique. Kumar et al. [23] reported on a comparative microstructural and mechanical property study between shot peening and vibro peening of nickel-based superalloys when subjected to the same peening intensity and observed that vibro peening generates deeper compressive residual stress as compared to shot peening at the same peening intensity (4–5 A) with good thermal stability at an elevated temperature. The CRS, microhardness, full-width half maxima (FWHM), plastic strain, and GOS profiles are compared in shot peening and vibro peening, as shown in Figure 20. The elevated temperature fatigue, corrosion fatigue, and sulphidation experiments have not been explored. Therefore, further investigation on the microstructure of surface and high-temperature fatigue is required for a comprehensive understanding of the process.

### 5.3. Deep Cold Rolling (DCR)

In deep cold rolling (DCR), a hydrostatically controlled ball is pressed and rolled against the surface in order to induce severe plastic deformation in the subsurface region [192]. The magnitude of surface compressive residual stress is relatively low but deeper into the subsurface as compared to the SP process. This process is currently limited to treat mostly flat surfaces and is difficult to implement on intricate geometries [17]. The combinations of high percentage cold work, higher depth of compressive residual stress, and lower roughness can yield a material with excellent fatigue life which is approximately five times that of conventional treatment methods [193]. Deep cold rolling is highly beneficial if an elevated thermal and mechanical overload is not present. The high cold work generated by the deep cold rolling process can relax the compressive stress rapidly.

Wong et al. [17] conducted DCR experiments on Ti-6Al-4V with three different designs of tools to show the feasibility of peened complex features. The maximum compressive residual stress reported was 1275 MPa magnitude within 1 mm. The process parameters, such as feed rate and overlapping, played a very insignificant role on the residual stress in the material as well the residual stress was higher consistently in the lateral direction.

Wagner et al. [194] studied the effect of DCR on the behavior of fatigue crack initiation and propagation of the aluminum, titanium, and magnesium alloys and illustrated that the cold work contributed to the retardation of crack nucleation but accelerated the propagation. However, compressive residual stress had little effect on the initiation but impeded the propagation of the cracks, whereas, surface roughness accelerated the crack initiation due to stress concentration. Nikitin et al. [195] studied the subsurface microstructure and cyclic deformation behavior of shot peened and deep rolled austenitic stainless steel AISI 304 under varying pressure and intensity. The complex microstructural features included deformation bands, nanocrystalline layer, and strain induced twins after both surface treatments. Thus, these features are highly responsible for the cyclic creep deformation behavior.

**Figure 21:**
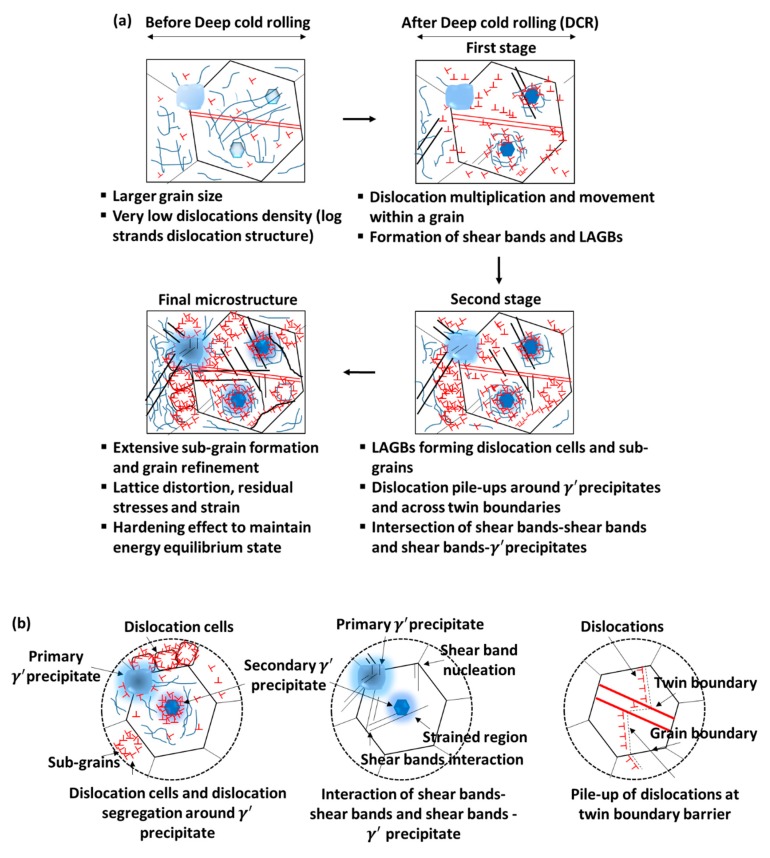
Schematic of (**a**) proposed microstructural evolution and strengthening mechanism during the DCR process and (**b**) effect of individual microstructural feature (adapted with permission from [22]).

Nalla et al. [16] investigated the effect of DCR and laser peening on the fatigue of the Ti-6Al-4V at a temperature range of ambient to 450 °C. The residual stress completely relaxed at 450 °C thermal exposure, but the benefit of the surface treatment was due to the generation of work hardening layer and nanocrystalline layer formation on the subsurface that impeded the driving force for fatigue failure. Recently, Kumar et al. [22] and Nagarajan et al. [196,197] presented the residual stress distribution, microstructural evolution, and proposed deformation and strengthening mechanisms induced during deep cold rolling of nickel-based superalloy Udimet 720Li under different hydrostatic pressure levels (10–50 MPa). Deformation induced defects, such as dislocation cells, shear bands, and their interactions, were observed during the DCR process, as presented in Figure 21. The plastic deformation during DCR was predominantly driven through the slip and dislocation multiplication mechanism.

### 5.4. Laser Shock Peening (LSP)

**Figure 22:**
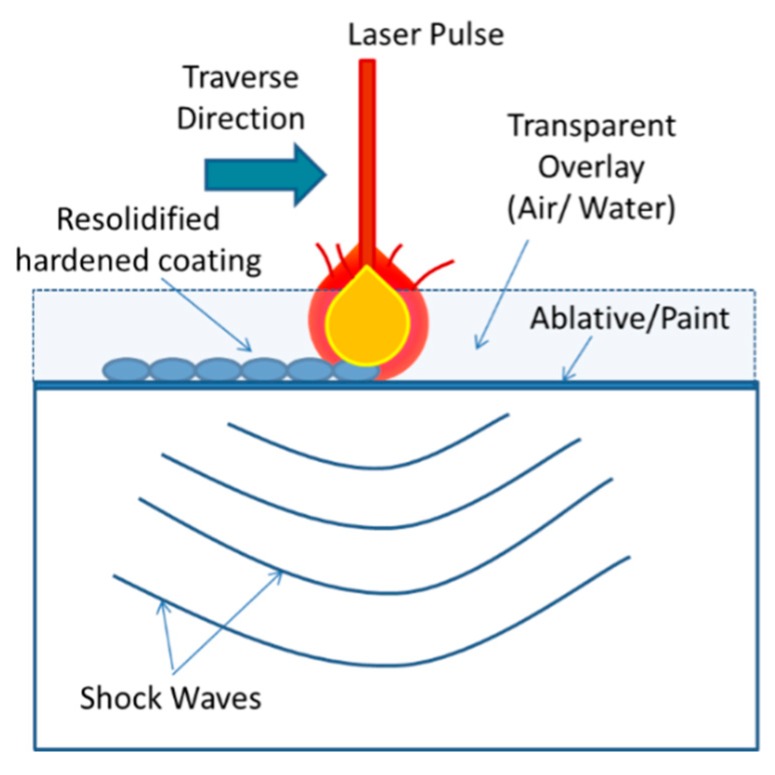
Schematic diagram of LSP (adapted with permission from [199]).

Laser shock peening (Figure 22) induces a high magnitude residual stress deep in the material by maintaining the low surface roughness [198] which is beneficial for surface-related failure such as fretting wear, fatigue, and stress corrosion cracking. High energy pulsed laser shock waves have been used for the generation of compressive residual stress. Nanosecond pulsed laser strikes on a transparent overlay (water, oil) confine the pulse energy on the metal surface and generate the high-pressure plasma. This plasma utilizes the mechanical effect of shock waves rather than thermal effects to deform the target elastically and plastically. Additionally, an absorbent coating with low impedance on the surface can further boost the intensity of shock waves by absorbing the pulse energy and provides interaction with material surface directly after expanding. Elastic deformation, which is under the Hugoniot elastic limit of deformation, regains its position and the rest plastic deformation causes the compressive residual stress and cold work in the material [199].

The LSP process parameters can be optimized to generate a higher volume of compressive stress with constraints on the depth of their influence and on the magnitude of tensile stress [200,201]. As the shock wave propagates through the material, its magnitude decreases according to the attenuation rate, which yields more compressive stress at the surface and a relatively decreased value towards the depth. The effects of laser peening parameters, i.e., fluence, coverage, repetition rate, etc. were studied by several researchers [21,202,203,204,205]. Smith et al. [206] studied the effect of power density and repetition rate on Ti-6Al-4V. The main conclusion was that power density has no effect on the residual stress and cold work on the given conditions, but the number of pulses per spot has an effect on both residual stress and cold work. The residual stress magnitude is high enough (−650 MPa) at the surface and the subsurface. The surface stresses (most likely the result of machining stresses) decayed to a minimum value (0 to −250 MPa) at the nominal mid-thickness of the section (i.e., ∼0.40 mm). Compressive residual stresses (CRS) and percentage cold work are both analogs to each other.

LSP is widely used for fatigue life improvement by inducing residual stress deep into the material and optimum microstructural features as reported by several researchers [21,89,207,208]. Prevey et al. [209] revealed the effect of the percentage cold work on the stress relaxation behavior of the IN718 material. The main conclusion was that laser peening and ball burnishing offer minimal percentage cold work, thus providing better resistance to thermal relaxation at elevated temperatures. Nikitin et al. [195] reported that the cycle, stress amplitude and temperature-dependent relaxation of compressive residual stress is more pronounced than the decrease of near-surface work hardening after thermal exposure in austenitic stainless steel AISI 304. However, gradual stress relaxation was observed for steels above 450 °C [210].

Peyre et al. [211] studied the high cycle fatigue performance of laser peened aluminum alloys. The major effect observed from other technology was that the depth of penetration of residual stress is approximately 1 mm, by far above any other process. The shortcoming of the LSP process, on other hand, was the hardening region that was 10% increment from the unpeened material and almost half of the conventionally peened material. Yang et al. [212] illustrated the effect of laser peening on the aluminum alloys fatigue performance with a fastener hole, multiple crack stop holes, and single-edge notch. The process was performed in a confined ablation mode using a Neodymium (Nd):glass laser at 5 GW cm^−2^ power density. The compressive residual stress generated due to shock waves was 385 MPa magnitude in the subsurface leading to good fatigue life with improved surface roughness. Tenaglia et al. [213] claimed that the LSP produced a number of beneficial effects for Ti-6Al-4V such as high fatigue life, and higher resistance towards fretting wear and stress corrosion cracking than the shot peening process due to the relatively high magnitude of compressive residual stress generated in LSP.

**Figure 23:**
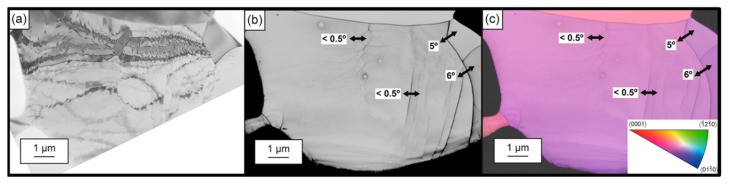
(**a**) BF-TEM micrograph of subgrains in the LSP sample, (**b**) TKD EBSD image, and (**c**) TKD EBSD inverse pole figure image showing low angle subgrain boundaries (adapted from [156]).

Lainé et al. [156] investigated the microstructural features of laser peened Ti-6Al-4V material and found complex microstructures such as numerous shears bands, low angle subgrain boundaries, and a few nanotwins (Figure 23). The directional arrays of planar dislocations with cellular structure and small subgrains with low angle grain boundaries were noticed. Several other researchers have also reported the same microstructural features after laser peening on different materials [207,214,215,216]. Dislocation density was increased significantly in laser peened aluminum alloys such as welded 5086-H32, 6061-T6 [217], 2024-T62 [218], and low carbon steel [219], but no quantitative analysis was conducted. Strain hardening in the material was comparatively less and caused low-stress relaxation at an elevated temperature. Lack of fundamental understanding of the laser peening literature has been noted for the interaction of the atomic or molecular structure with laser-induced shock waves and the resulting changes in the microstructure.

### 5.5. Water Jet Cavitation Peening (WJCP)

Water jet cavitation peening (WJCP) uses a high-velocity water jet to impact the surface of a material for multiple processes such as surface cleaning, paint removal, and cutting [220,221,222]. Water jet cavitation peening (WJCP) or water peening (WP) is similar to SP except that it uses high-pressure droplets that disintegrate in the water jet flow field instead of solid shots. There are other similar concepts of peening like water droplet peening, water jet cavitation peening in water or air, and oil jet peening [223]. The water peening (WP) process is a physically complex technique.

A comparative study of shot peening and cavitation shot peening (CSP) on carburized steel was done by Odhiambo et al. [224]. Surprisingly, the residual stress was 1189 MPa in SP with a 7% increase in fatigue life from pristine sample, whereas, CSP produced only 560 MPa compressive residual stress (CRS), but with an improved fatigue life of 11%. Furthermore, Taguchi optimization of the process identified the influencing factors responsible for higher fatigue life. It was observed that cavitation number played a significant role in residual stress and surface roughness while nozzle size had a larger impact on surface roughness. A higher cavitation number yielded better results for improving the fatigue strength of chrome-molybdenum steel.

Grinspan et al. [223] peened AISI 1040 medium carbon steel with oil jet, which led to 390 MPa compressive residual stress and 332 MPa distribution up to 50 μm with 20% improvement in fatigue life. The effects of peening by various oils on a range of engineering alloys was carried out by Pai et al. [225]. It was observed that vegetable oil exhibits the best medium for erosion resistance for all metals due to its high viscosity index and materials with high hardness had less cavitation damage for all lubricants [225]. Soyama et al. [226] reported a 50% improvement in fatigue life for aluminum alloys when subjected to cavitation shotless peening using a 30 MPa plunger pump. The pits resulting from cavitation shotless peening were of various sizes from 20 µm to 100 µm, giving a compressive residual stress of 200 MPa with a surface roughness of 0.22 µm. Han et al. [227] conducted water cavitation with aeration on SAE 1050 steel, and measured 215 MPa residual stress which gradually reduced to zero at a depth of 110 µm. The samples were quenched leading to an increase in residual stress up to 535 MPa to a depth of 140 µm. Hence, post processing of WJCP samples further improved the fatigue life. Several other researchers [152,228,229,230,231] reported a fatigue life improvement of 40–60% with variations of process parameters and medium due to compressive residual stress.

In another study, Ju et al. [145] investigated the microstructural features after WJCP treatment of pure titanium and reported that the twinning generated by the treatment played a vital role in plastic deformation and residual stress of hexagonal structure. The deformation twinning, twinning interaction, and high dislocation density in the strain hardening region were observed which could be responsible for the stability of residual stress [232].

### 5.6. Ultrasonic Shot Peening (USSP)

The principle of ultrasonic shot peening (USSP) is based on the vibration of spherical shots using a high-power ultrasound at high frequency. The vibrations shot peen the surface by repeated impacts over a short period. The main parameters of the USSP process are the vibration frequency of the chamber driven by an ultrasonic generator, shot diameter, and processing time [232].

Liu et al. [232] used USSP to treat 316L stainless steel and studied the effect of processing duration 30 s to 810 s on the microstructure. In all cases, a grain refinement in the surface layer and nanocrystalline layer of 30 µm thickness was observed. Similarly, the same deformation behavior and thickness were obtained in various types of steels [233] and aluminum alloys [234]. Villegas et al. [235] and [236], with WC/Co balls of 7.9 mm diameter for USSP, reported that nanocrystalline layers obtained on Ni-base C-2000 alloy increased with the processing time. The grain refinement occured due to deformation twin and was complemented by the dislocation activity, which justified the generation of nanocrystalline. The grain refinement induced, and the work-hardening associated with the USSP process itself led to a surface hardening. The hardness increment occurred in 16MnCr5 steel when displacement amplitude increased and the distance between nozzle and sample decreased [237]. Hou et al. [238] also reported high surface hardness when the grain size was very small (approximately 20 nm) on a magnesium alloy surface and 200 nm in bulk material, due to the generation of the nanocrystalline layer.

Xing et al. [239] investigated the effect of the USSP process on the fatigue life of steel. A compressive residual stress (309 MPa) to a depth of 300 µm was developed in the subsurface and improved the total strength, stiffness, and fatigue life of the material. The equiaxed nanocrystals with a grain size of a few nanometers (i.e., 10 nm) were observed in the subsurface layers. The grain refinement mechanism could be related to the activity of the high density of dislocations and the formation of small shear bands. A similar trend of results has been published by several other researchers on iron [240,241] and stainless steel [232,242].

However, as Dai et al. [243] reported, the surface roughness generated by the indentation process of the bombarding balls may induce stress concentration at specific sites, which may result in crack initiation and fatigue failure. To avoid these cracks, protective coating must be applied during the peening process. These coatings can also absorb the energy and induce a greater concentration of energy on the surface or they can also impede the impact effect on the surface. Various coatings provide a different role in surface topography and properties.

**Figure 24:**
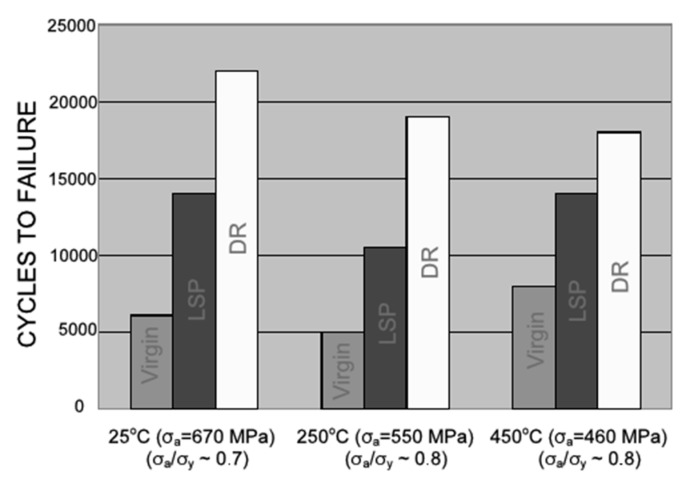
Enhancement in fatigue lifetimes following deep rolling and laser shock peening for test temperatures of 25, 250, and 450 °C and stress amplitudes of 670, 550, and 460 MPa, respectively, for Ti-6Al-4V (adapted with permission from [16]).

Overall, a comparative study was conducted for residual stress and fatigue performance based on the experimental data published for various materials and processing parameters. For example, deep cold rolling (DCR) or deep rolling (DR) generated a relatively high magnitude of residual stress deeper in the subsurface than LSP and, hence, showed a higher cycle to failure (as shown in Figure 24) at various temperatures for given input parameters [16]. In LSP, the power density used was 7 GWcm^−2^ with 200% coverage and for DCR, 500 N hydrostatic force with 1000% coverage.

**Figure 25:**
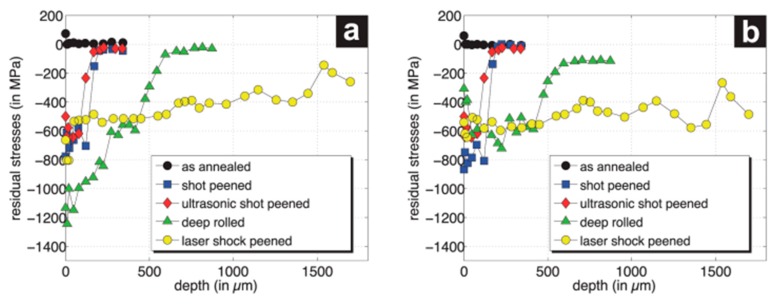

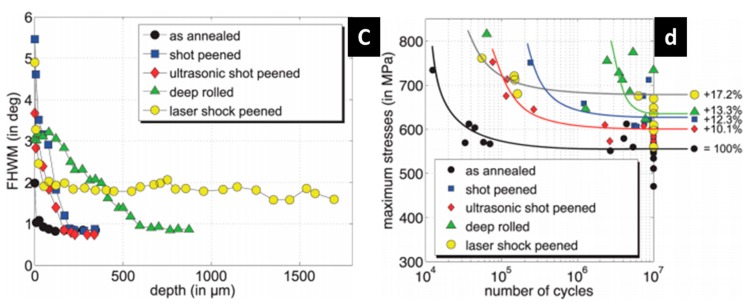
Comparison of Ti-6Al-4V for SP, USSP, DCR, LSP (**a**) axial, (**b**) tangential residual stress profiles, (**c**) FWHM, and (**d**) maximum stress for failure (adapted with permission from [144]).

Residual stress depth of influence is higher in LSP followed by DCR, USSP, SP, in both the axial and the tangential direction (as shown in Figure 25a,b) at the given input process conditions. However, the residual stress magnitude is higher in DCR followed by LSP, SP, and USSP axial direction. In the tangential direction, SP residual stress magnitude is higher followed by LSP, USSP, and DCR. Furthermore, full-width half maxima (FWHM) is higher in SP followed by LSP, USSP, DCR, as shown in Figure 25c. SP at Almen intensity 0.012” A with 200% coverage was used for generating the deformation, and 30 MPa hydrostatic pressure with 530 rpm and 0.1 mm/rev was used for the DCR process. In USSP, a peening intensity of F20.12 A with 80 µm amplitude was applied for 120 s to maintain more than 125% coverage. In LSP, a high energy laser with power density 10 GWcm^−2^ and 8 ns pulse length with 100% coverage was applied.

A comparison study of different surface treatments revealed that LSP generates a relatively higher fatigue life (HCF) followed by DCR, SP, and USSP for Ti-6Al-4V for given input parameters, as shown in Figure 25d. The values are presented based on the experimental evidence. The fatigue life increment was calculated based on the life extension after surface treatment with reference to respective untreated condition. In another study by Maawad [244], it was reported that HCF performance of Ti-2.5Cu was superior after ball burnishing as compared to laser peening without coating or the shot peening process. Therefore, a comprehensive study on different materials with a variety of surface treatments and different processing parameters is required to confirm the effectiveness of each surface treatment process.

## 6. Major Conclusions and Scope for Future Research Work

From the above discourse, the effects of microstructure on the mechanical properties of engineering alloys are listed in Table 2. The following broad conclusions can be made from this literature review:

1. To achieve a good combination of strength and ductility simultaneously, gradient microstructure, i.e., nanocrystalline on the surface and coarse in core can be produced in the material using surface mechanical treatments.
2. Residual compressive stress and strain hardening are beneficial for improving fatigue crack resistance. However, strain hardening is adversely affected at elevated temperatures due to dominance of creep.
3. For elevated temperature applications, stress relaxation behavior is a recurrent phenomenon because of the dislocation annihilation mechanism, but it can be controlled by resisting the atomic motions on slip planes by introducing twin grain boundaries.
4. Surface mechanical treatments with varying strain rates could generate grain size, residual stress, strain hardening, and other defects with varying distribution throughout the material depth. Additionally, optimization is essential for developing material with excellent performance.
5. For surface treatments, LSP shows relatively high fatigue performance and deeper residual stress as compared with SP, USSP, WJCP, and DCR for the studied process parameters. However, the same input energy, coverage, and material is required for a better comparison of the processes and performance. Hybrid processes like shot peening followed by deep cold rolling could be beneficial for the fatigue life, as a high magnitude of compressive residual stress deeper in the material can be achieved.

The role of individual factors is explained here:

1. **Grain Size**: Decreasing grain size increases strain hardening as per the Hall–Petch relationship, thus, resistance to crack initiation increases. Additionally, decreasing grain size increases the frequency of crack encounters boundaries, which provides more resistance to the crack growth.
2. **Grain Distribution**: Surface nanograin structure strengthens the material while interior coarse grains maintains the ductility of the material. The hard-and-deformed nanograined surface structure suppresses the crack initiation while the soft coarse-grain interior structure is effective in arresting the cracks [53]. Strain delocalization in gradient nanostructure is responsible for enhancing fatigue resistance in cyclic loading/unloading.
3. **Twin GBs**: Twin boundaries toughen the material by the dislocation-twin interaction that provides a dislocation nucleation site. When dislocations strike with twin boundaries, stress concentration occurs at GBs. To eliminate stress localization, dislocation nucleation takes place on another side of the grain boundary. This dislocation nucleation and pile-ups make twin boundary a source of dislocation generation, thus, improving the toughness of the material.
4. **High Angle GBs**: High angle GBs with ultrafine grain and nonequilibrium structure encourage grain boundary sliding, and thus, improve the ductility of the material. Simultaneously, ultrafine grain strengthens the material. Thus, a combination of high strength and ductility can be achieved through gradient microstructure.
5. **Low Angle GBs**: Low angle GBs are generally poor in grain boundary sliding which leads to lower ductility and improves the hardness of the material significantly.
6. **Dislocations**: Resistance to dislocation movement provides the strengthening of the material. However, dislocation pile-ups at the grain boundaries or precipitates generate the stress concentration, and therefore dislocation either moves to another grain to reduce the stress intensity or initiates the cracks at the grain boundaries.
7. **Strain Hardening**: Strain hardening effect, generated due to dislocation nucleation and pile-up, increases the hardness but reduces the ductility of the material. The influence of strain hardening is still ambiguous as both beneficial and adverse effects on fatigue life have been reported by several researchers. The higher effective depth of the strain-hardened region rather than its high magnitude is anticipated for higher mechanical performance.
8. **Compressive residual stress**: Compressive residual stress compensates the tensile stress generated due to applied load that reduces the chances of crack initiation on the surface. Compressive residual stress and stress gradient throughout the depth are beneficial for fatigue life as they can provide high resistance towards both crack initiation and propagation mechanism. There are still arguments whether the high depth of influence or high magnitude of compressive residual stress is beneficial for high fatigue performance.

## Figures and Tables

**Table 1:** Comparison of fatigue life enhancement by various microstructural features.

Material	SP Parameters	Microstructural Features	Fatigue Life
AISI 9310 Steel [189]	Intensity: 0.18–0.23 mm A, coverage: 200%, media: cast steel 070	40% increment in CRS for SP gear at depth of maximum shear stress	10 percent surface pitting fatigue life increased by 1.6 times than standard gear (unpeened)
Bearing steel (JIS-SUJ2) [190]	Media: 0.3 mm diameter, coverage:300% coverage	3.75 times higher RS (−1530 MPa) up to 90 µm, 1.18 times higher Vickers hardness (1019 HV)	6–7 times increment after treatment in bending fatigue, 0.3% in ultimate tensile strength
51CrV4 high-grade spring steel alloy [56]	Media: 0.8–0.9 mm diameter shots, 0.3% intensity, 100% coverage	The maximum measured stress of 1100 MPa arises at a distance of 210 mm measured from the specimen’s middle point	The decrease in 30% sustainable stress amplitude due to high surface roughness
Low alloy steel [191]	Intensity: 15 A (CSP),100%,0.42 mm diameter 7 C (SSP), 1500% coverage, 0.58 mm diameter, (60–61 HRC) 10 N (RSSP),100%, 0.10 mm diameter	0.3–0.35 mm thick strain hardening depth, 0.65 (CSP), 1.38 mm (SSP), −580 MPa in both, observed NC nanocrystalline layer using TEM	4% increment after SSP and 10% after repeening SSP on fatigue strength
Nickel-based superalloy RR1000 [97]	Intensity:6–8 A, media: 110 H, coverage: 200%	CRS: (1100 S, 1400 M) MPa, up to 200 µm depth Stress relaxation: (400 S, 900 M) MPa, up to 200 µm depth, peak shifted from 50 to 75 µm SH: strain hardening depth (100–125 µm), 21% relaxation in percentage hardening	Dwell fatigue relax the compressive residual stress and strain hardening in the material in the first few cycles
Nickel-based superalloy Udimet 720Li [23]	Intensity: 4–5 A, media: 110H, coverage: 125%	CRS: (826 S, 1094 M) MPa up to depth of 140 μm, 30% increment in Vickers microhardness	-
AISI 4340 steel [162]	Intensity: range [0.0027 A (8 psi), 0.0063 A (13 psi), 0.0083 A (18 psi), 0.0141 A (45 psi)], coverage: 200%, media: S 230 (0.7 mm diameter)	CRS:1200 MPa up to 0.175 mm depth	9–12% increment in fatigue life

**Table 2:** Effect of microstructural features on properties and fatigue crack mechanism.

Features	Specifications	Properties	Crack Initiation	Crack Propagation	Material Specifications
**Grain size**	Ultrafine or nanocrystalline (↑)	Strength (↑) Hardness (↑) Ductility (↓)	Retards	Retards, or Accelerate both reported	Brass [245], Ti [246], stainless steel [247] Electrodeposited nanocrystalline pure Ni and a cryomilled ultrafine-crystalline Al–Mg alloy, Ni films [248]
**Grain distribution**	Gradient nanostructure or gradient nanotwined structure (↑)	Strength (↑) Ductility (↑)	Retards	Retards	Cu [50,53], steel [249] (torsion to cylindrical twinning-induced plasticity steel to generate gradient nanotwinned structure)
**Twin GBs**	Nanotwinned or coherent nanotwin boundaries (↑)	Strength (↑) Ductility (↑) Toughness (↑) [250]	-	Retards	Cu [250] (CLSP)
**Low angle GBs**	Misorientation angle <15° (↑)	Ductility (↓) Hardness (↑)	-	-	All polycrystalline metals
**High angle GBs**	Misorientation angle ≥15° (↑)	Ductility (↑) Hardness (↓)	-	-	HAGB with UFC and nonequilibrium GBs [57]
**Dislocations**	Generation and pile-ups ()	Strength (↑) Hardening (↑) Ductility (↓)	-	-	Nanocrystalline/ultrafine structure [57]
**Strain hardening**	Magnitude and distribution depth (↑)	Hardness (↑) Ductility (↓)	Retards	Retards and accelerate (both reported)	Polycrystalline materials
**Compressive residual stresses**	Compressive stresses or distribution depth (↑)	Hardness (↑) (little influence)	Retards	Retards (little effect)	Polycrystalline materials
